# Kindlin-2 regulates skeletal homeostasis by modulating PTH1R in mice

**DOI:** 10.1038/s41392-020-00328-y

**Published:** 2020-12-26

**Authors:** Xuekun Fu, Bo Zhou, Qinnan Yan, Chu Tao, Lei Qin, Xiaohao Wu, Sixiong Lin, Sheng Chen, Yumei Lai, Xuenong Zou, Zengwu Shao, Meiqing Wang, Di Chen, Wenfei Jin, Youqiang Song, Huiling Cao, Ge Zhang, Guozhi Xiao

**Affiliations:** 1 School of Medicine, Guangdong Provincial Key Laboratory of Cell Microenvironment and Disease Research, and Shenzhen Key Laboratory of Cell Microenvironment, Southern University of Science and Technology, 1088 Xue Yuan Road, 518055 Shenzhen, Guangdong China; 2 Law Sau Fai Institute for Advancing Translational Medicine in Bone and Joint Diseases, School of Chinese Medicine, Hong Kong Baptist University, 999077 Hong Kong, SAR China; 3 Department of Biology, Southern University of Science and Technology, 518055 Shenzhen, China; 4 School of Biomedical Sciences, University of Hong Kong, 21 Sassoon Road, Hong Kong, China; 5 Department of Spine Surgery, Orthopedic Research Institute, The First Affiliated Hospital of Sun Yat-sen University, Guangdong Provincial Key Laboratory of Orthopedics and Traumatology, 510080 Guangzhou, China; 6 Department of Orthopedics, Union Hospital, Tongji Medical College, Huazhong University of Science and Technology, 430022 Wuhan, China; 7 Department of Orthopedic Surgery, Rush University Medical Center, Chicago, IL 60612 USA; 8 State Key Laboratory of Military Stomatology, National Clinical Research Center for Oral Diseases, Shanxi International Joint Research Center for Oral Diseases, Department of Oral Anatomy and Physiology and TMD, School of Stomatology, the Fourth Military Medical University, 145 Changle West Road, Xi’an, China; 9 Research Center for Human Tissues and Organs Degeneration, Shenzhen Institutes of Advanced Technology, Chinese Academy of Sciences, 518055 Shenzhen, China

**Keywords:** Metabolic disorders, Cell biology

## Abstract

In vertebrates, the type 1 parathyroid hormone receptor (PTH1R) is a critical regulator of skeletal development and homeostasis; however, how it is modulated is incompletely understood. Here we report that deleting Kindlin-2 in osteoblastic cells using the mouse 10-kb *Dmp1-Cre* largely neutralizes the intermittent PTH-stimulated increasing of bone volume fraction and bone mineral density by impairing both osteoblast and osteoclast formation in murine adult bone. Single-cell profiling reveals that Kindlin-2 loss increases the proportion of osteoblasts, but not mesenchymal stem cells, chondrocytes and fibroblasts, in non-hematopoietic bone marrow cells, with concomitant depletion of osteoblasts on the bone surfaces, especially those stimulated by PTH. Furthermore, haploinsufficiency of *Kindlin-2* and *Pth1r* genes, but not that of either gene, in mice significantly decreases basal and, to a larger extent, PTH-stimulated bone mass, supporting the notion that both factors function in the same genetic pathway. Mechanistically, Kindlin-2 interacts with the C-terminal cytoplasmic domain of PTH1R via aa 474–475 and Gsα. Kindlin-2 loss suppresses PTH induction of cAMP production and CREB phosphorylation in cultured osteoblasts and in bone. Interestingly, PTH promotes Kindlin-2 expression in vitro and in vivo, thus creating a positive feedback regulatory loop. Finally, estrogen deficiency induced by ovariectomy drastically decreases expression of Kindlin-2 protein in osteocytes embedded in the bone matrix and Kindlin-2 loss essentially abolishes the PTH anabolic activity in bone in ovariectomized mice. Thus, we demonstrate that Kindlin-2 functions as an intrinsic component of the PTH1R signaling pathway in osteoblastic cells to regulate bone mass accrual and homeostasis.

## Introduction

The type 1 parathyroid hormone receptor (PTH1R), a G-protein-coupled seven transmembrane receptor (GPCR), is the primary functional receptor for both endogenous PTH and PTH-related protein (PTHrP) ligands. PTH1R plays critical roles in regulation of calcium metabolism, skeletal development, and homeostasis.^1–10^ Abnormalities in PTH/PTHrP/PTH1R signaling cause human diseases, such as hypercalcemia, osteoporosis, tumorigenesis, and metastasis.^11,12^ Therefore, it is important to understand how PTH1R is regulated under physiological and pathological conditions.

Intermittent administration of PTH, a treatment for severe osteoporosis approved by the United States Food and Drug Administration, increases the bone mass and bone mineral density (BMD) and improves bone microstructure in a number of animal models and in humans.^13^ PTH exerts its anabolic activity in bone through the action of PTH1R that is primarily expressed in cells of the osteoblastic lineage. PTH promotes the mesenchymal stem cell (MSC) differentiation into osteoblasts and subsequent osteoblast proliferation and differentiation and inhibits osteoblast apoptosis.^6,14–17^ Osteocytes are terminally differentiated osteoblasts. It is now believed that osteocytes orchestrate the bone remodeling by synthesizing and secreting crucial factors, such as sclerostin and RANKL.^18–20^ Cumulative evidence suggests that osteocytes might mediate the PTH anabolic activity in bone.^21,22^ A better understanding of how intermittent PTH functions will help develop strategies to improve its treatment and avoid its potential side effects, such as reduced therapeutic effect after long-term application, hypocalcemia, hypercalcemia, and osteosarcoma.

Kindlin-2 is a key focal adhesion protein that activates integrin and promotes cell-extracellular matrix (ECM) adhesion and migration.^23^ Recent studies from our group and others have uncovered important roles of Kindlin-2 in regulation of organogenesis and homeostasis of skeleton, kidney, adipose tissue, heart, pancreas, and intestine.^24–32^ Zhang et al. reported that Kindlin-2 plays an essential role in preserving integrity of the developing heart and preventing ventricular rupture in mice.^27^ We demonstrated that Kindlin-2 regulates skeletogenesis by modulating TGF-β signaling and Sox9 expression in MSC and chondrocyte.^24^ We further demonstrated that Kindlin-2 determines whether MSC differentiates into osteoblasts or adipocytes through control of Yap1/Taz.^33^ More recently, we demonstrated that Kindlin-2 regulates bone remodeling in mice through modulation of expression of sclerostin and Rankl in osteocytes.^30^

Using multiple genetically modified mouse models combined with single-cell profiling and biochemical approaches, in this study, we establish that Kindlin-2 and PTH1R cooperatively regulate basal and intermittent PTH-stimulated bone mass accrual and homeostasis.

## Results

Kindlin-2 loss in osteoblastic cells severely impairs intermittent PTH-stimulated increases in bone volume faction and BMD by impairing both osteoblast and osteoclast formation in male and female adult mice.

**Fig. 1:**
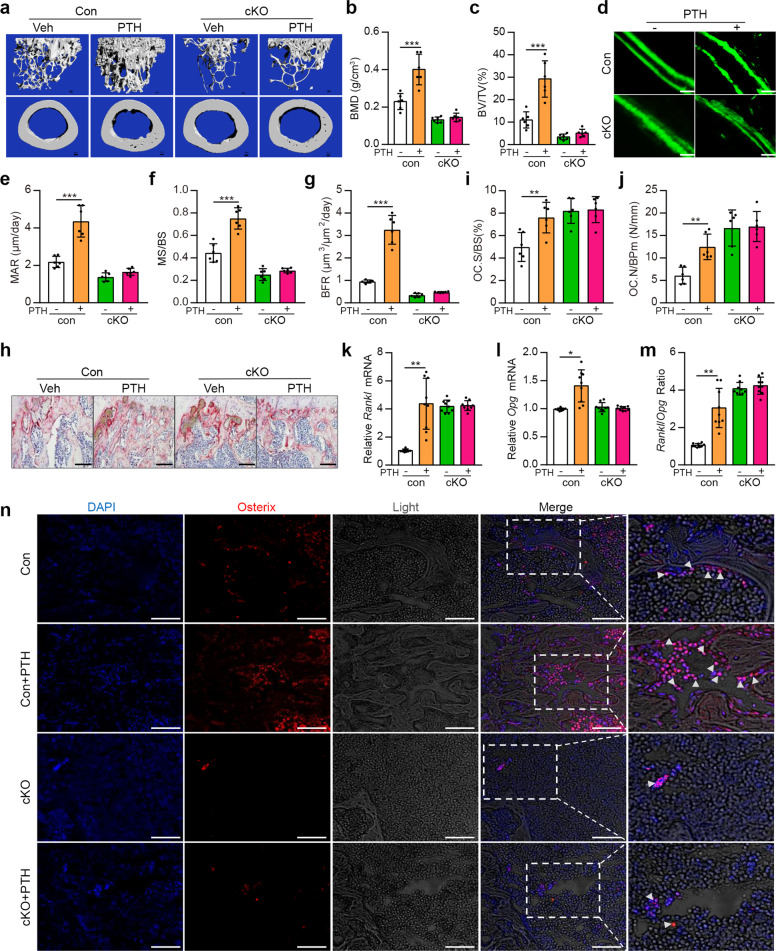
Kindlin-2 loss in osteoblastic cells severely impairs skeletal response to intermittent PTH by affecting osteoblast and osteoclast function. **a** Three-dimensional (3D) images of micro-computerized tomography (μCT) of distal femurs from 3-month-old control (Cre-negative *Kindlin-2*^*f/f*^) and *Dmp1-Cre; Kindlin-2*^*f/f*^ (cKO) female mice with and without PTH treatment for 28 d starting at the age of 3 months. **b**, **c** Quantitative analyses of the bone mineral density (BMD) and bone volume/tissue volume (BV/TV). *N* = 6 per group for both control and cKO. Results are expressed as mean ± standard deviation (s.d.). *P < 0.05, **P < 0.01, ***P < 0.001 (**a**–**m**). **d**–**g** Calcein double labeling. Images of the mineralized surface of the non-demineralized distal femoral sections (**d**). Scale bars: 20 μm. Quantitative analyses of measurement of the mineral apposition rate (MAR) (**e**), mineralizing surface per bone surface (MS/BS) (**f**), and bone formation rate (BFR) (**g**). *N* = 6 per group for both control and cKO. **h**–**j** Osteoclast formation in bone. Tibial sections of **a** were subjected to tartrate-resistant acid phosphatase (TRAP) staining. Scale bars: 50 μm. Quantitative analyses of the osteoclast surface/bone surface (Oc.S/BS) (**i**) and osteoclast number/bone perimeter (Oc.N/BPm) (**j**). *N* = 6 per group for both control and cKO. **k**–**m** Real-time RT-PCR (qPCR) analysis. Total RNA isolated from above control cKO bones was used for qPCR analysis for expression of *Rankl* and *Opg* mRNA, which was normalized to *Gapdh* mRNA. Experiments were independently repeated three times. **n** Immunofluorescence (IF) staining. Sections of tibial sections were subjected to IF staining with an antibody against osterix (Osx). Scale bars: 50 μm. Arrowheads indicate Osx-expressing osteoblasts

To determine whether Kindlin-2 plays a role in mediation of the anabolic effects of intermittent PTH on bone, we deleted its expression in osteoblastic cells using the 10-kb *Dmp1-Cre* transgenic mice and determined its impact on the PTH effects on bone. To avoid potential effects of animal rapid growth during skeletal development on the PTH effects, we utilized 3-month-old adult mice, which have mature skeleton, for this experiment. We used Cre-negative *Kindlin-2*^*fl/fl*^ mice as controls. Control and *Dmp1-Cre; Kindlin-2*^*f/f*^ mice (referred to as cKO hereafter) female mice were subcutaneously injected with daily PTH 1-34 (100 μg/kg body weight) for 28 d as we previously described.^34^ Mice were sacrificed 24 h after the last PTH injection. X-ray micro-computed tomography (μCT) analyses of distal femurs revealed that the PTH-stimulated increases in bone volume and BMD in control mice were dramatically decreased in cKO mice (Fig. 1a–c). Specifically, PTH increased the BMD, bone volume fraction (BV/TV), and trabecular number (Tb.N) by 75.1%, 166.1%, and 126.2%, respectively, and decreased the trabecular separation (Tb.Sp) by 27.3% in control mice (Fig. 1b, c and supplementary Fig. 1a, b). Strikingly, the PTH-induced alterations were dramatically reduced (BV/TV and Tb.N) or completely lost (BMD and Tb.Sp) in cKO mice. Notably, this PTH regimen did not significantly increase the trabecular thickness (Tb.Th) and cortical thickness (Cort.Th) in both genotypes (supplementary Fig. 1c, d). Collectively, these results clearly demonstrate an essential requirement for Kindlin-2 in mediating the anabolic effects of intermittent PTH on bone.

Because it is known that intermittent PTH exerts its anabolic activity in bone by primarily targeting the osteoblastic lineage cells, we measured the bone-forming activity of osteoblasts in vivo by performing the double calcein labeling experiments. As expected, we observed significant increasing of the mineralization apposition rate (MAR), mineralizing surface per bone surface (MS/BS) and bone formation rate (BFR) after PTH treatment in control mice. Strikingly, these PTH-stimulated changes in osteoblast parameters were dramatically decreased (MAR and BFR) or completely lost (MS/BS) in cKO mice (Fig. 1d–g).

Results from the tartrate-resistant acid phosphatase (TRAP) staining of tibial sections showed that PTH treatment promoted the osteoclast formation, as demonstrated by the increasing of the osteoclast surface/bone surface (Oc.S/BS) and osteoclast number/bone perimeter (Oc.Nb/BPm) in control bones (Fig. 1h–j). While Kindlin-2 loss increased the basal osteoclast formation, it completely abolished PTH-stimulated increase in osteoclast formation in bone (Fig. 1h–j). PTH increased the *Rankl*/*Opg* ratio by dramatically increasing the expression of *Rankl* mRNA, but not as much as of the *Opg* mRNA in control mice, and these PTH-induced changes were barely detectable in cKO bones (Fig. 1k–m).

Consistent with reduced MAR and BMR, results from immunofluorescence (IF) staining of bone sections revealed a number of osterix (Osx)-positive osteoblasts, which were primarily located on the trabecular bone surfaces. Few Osx(+) osteoblasts were observed in cKO bones (Fig. 1n). Furthermore, PTH dramatically increased the numbers of Osx(+) osteoblasts in control bone (Fig. 1n). However, this dramatic PTH effect was not observed in cKO bones (Fig. 1n).

### Single-cell profiling reveals that Kindlin-2 loss retains osteoblasts, but not MSCs, chondrocytes and fibroblasts, in the bone marrow and inhibits PTH stimulation of osteoblast gene expression

**Fig. 2:**
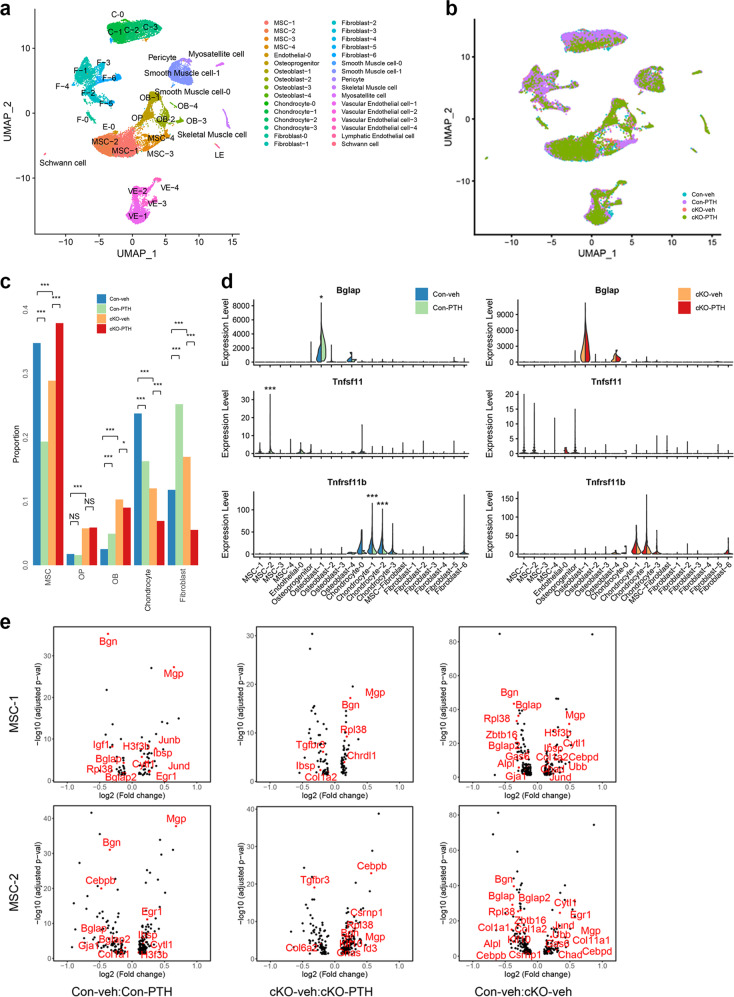
*Kindlin*-2 loss decreases BM osteoblast population and inhibits PTH stimulation of osteoblast differentiation. **a** UMAP visualization of cells. Clusters were marked with identified cell types. **b** UMAP visualization of cells, colored based on samples. **c** Proportions of selected cell types in each sample. *P* value of chi-square test was marked. **d** Violin plot of Bglap, Tnfsf11 and Tnfrsf11b in selected cell types. Expression levels were indicated by UMI counts. **e** Volcano plot of significantly and differentially expressed genes (Bonferroni corrected *p* value < 0.05) between samples under different conditions in MSC-1 and MSC-2. Ossification-related genes were marked as red color

We next determined the effects of Kindlin-2 loss on the bone marrow (BM) cells by performing single-cell RNA-sequencing (scRNA-seq) of BM non-hematopoietic cells from 3-month-old control and cKO mice with and without PTH treatment. We used the Lineage Cell Depletion Kit combined with flow cytometry to deplete the hematopoietic cells, such as T cells, B cells, monocytes/macrophages, granulocytes, and erythrocytes and their precursors, from pooled whole BM cells of each group. After that, the non-hematopoietic cells were processed based on 10x Genomics Chromium Single Cell 3’ protocol (v3 Chemistry). We profiled 23341 cells from the four groups, including 5065 cells from con-veh, 4853 cells from con-PTH, 7385 cells from cKO-veh and 6038 cells from cKO-PTH (Fig. 2a, b). We defined cell types for all clusters as the mesenchymal stem cell (MSC), osteoprogenitor (OP), osteoblast (OB), chondrocyte, fibroblast, smooth muscle cell, skeletal muscle cell, pericyte, myosatellite cell, vascular endothelial cell, lymphatic endothelial cell, and Schwann cell according to their gene expression patterns and relative expression levels of respective signature genes (supplementary Fig. 2a, b). To investigate whether the proportions of non-hematopoietic cells were affected by Kindlin-2 loss and/or intermittent PTH treatment, we determined the proportions of each cell type and cluster for all samples (Fig. 2c and supplementary Fig. 2d) and found that the proportions of several cell types, including MSC, OP, OB, chondrocyte, and fibroblast, were mostly affected by PTH and/or Kindlin-2 loss. For examples, Kindlin-2 loss significantly decreased the proportions of MSC and chondrocyte. In contrast, Kindlin-2 loss significantly increased the proportions of OP, OB, and fibroblast. This result along with above result (Fig. 1n) suggests that Kindlin-2 loss retains osteoblasts in the BM, which normally migrate onto the bone surfaces where they deposit mineralizing matrix and form bones. PTH treatment increased the proportions of OB and fibroblast but decreased those of MSC and chondrocyte in control bones (Fig. 2c). In contrast, PTH increased the proportion of MSC but decreased those of OB, chondrocyte and fibroblast in cKO mice. Differential gene expression analysis revealed that PTH increased the expression level of osteocalcin (*Bglap*), a marker for mature osteoblast, in Osteoblast-1 cluster of control but not cKO mice, while it increased that of *Rankl* (*Tnfsf11*), a key factor for osteoclast formation and differentiation, in MSC-2 cluster in control but not in that in cKO mice (Fig. 2d). Surprisingly, we found that osteoprotegerin (*Opg* or *Tnfrsf11b*), a potent inhibitor of the osteoclast formation and bone resorption, was mostly expressed in chondrocytes in BM cells and that its expression was decreased by PTH in control but not cKO group (Fig. 2d). Many significantly and differentially expressed genes were observed in clusters of MSC, especially in clusters MSC-1 and MSC-2 (supplementary Fig. 2c). Gene enrichment analysis showed that expression of ossification-related genes was upregulated by PTH in control but not cKO group (supplementary Fig. 2e). Among the ossification-related genes, insulin-like growth factor I (Igf1) was most significantly upregulated by PTH in control but not cKO group. In contrast, transforming growth factor beta receptor type 3 (Tgfbr3) was most significantly upregulated by PTH in cKO but not control group (Fig. 2e).

### Deleting one allele of Kindlin-2 and Pth1r genes, but not one allele of either gene, in osteoblastic cells decreases basal and, to a larger extent, PTH-stimulated bone volume and BMD in adult mice of both sexes

**Fig. 3:**
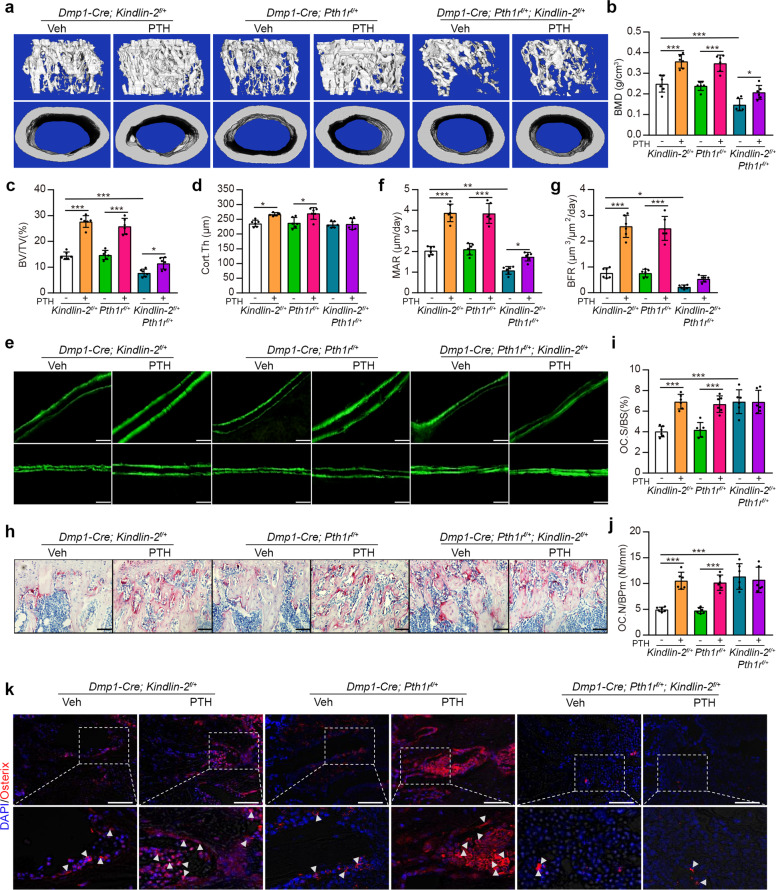
Haploinsufficiency of *Kindlin*-2 and *Pth1r* genes decreases basal and, to a larger extent, PTH-stimulated bone mass. **a** Three-dimensional (3D) images of μCT of distal femurs from *Dmp1-Cre; Kindlin-2*^*f/+*^, *Dmp1-Cre; Pth1r*^*f//+*^ and *Dmp1-Cre; Kindlin-2*^*f/+*^*; Pth1r*^*f//+*^ male mice with and without PTH treatment for 28 d starting at the age of 3 months. **b**–**d** Quantitative analyses of the bone mineral density (BMD), bone volume/tissue volume (BV/TV), and cortical thickness (Cort.Th) of distal femurs. *N* = 6 mice per group. Results are expressed as mean ± standard deviation (s.d.). *P < 0.05, **P < 0.01, ***P < 0.001 (**a**–**j**), veh versus PTH or single heterozygote versus double heterozygote. **e** Calcein double-labeling images of the mineralized surface of the non-demineralized distal femora. Scale bars: 50 μm. **f**, **g** Quantitative analyses of measurement of the mineral apposition rate (MAR) (**f**) and bone formation rate (BFR) (**g**). *N* = 6 mice per group. **h**–**j** Osteoclast formation. Quantitative analyses of the osteoclast surface/bone surface (Oc.S/BS) and osteoclast number/bone perimeter (Oc.N/BPm). *N* = 6 mice per group. **k** Immunofluorescence (IF) staining. Sections of tibial sections of the indicated groups were subjected to IF staining with Osx antibody. Scale bars: 50 μm. Arrowheads indicate Osx(+) osteoblasts

We next investigated whether there is a functional interplay between Kindlin-2 and PTH1R in bone. To this end, we deleted one allele of *Kindlin-2* and/or *Pth1r* genes in osteoblastic cells using the same *Dmp1-Cre* mice, and we generated *Kindlin-2* or *Pth1r* singly heterozygous mice (i.e., *Dmp1-Cre; Kindlin-2*^*f/+*^ or *Dmp1-Cre; Pth1r*^*f/+*^) and double heterozygous mice (i.e., *Dmp1-Cre; Kindlin-2*^*f/+*^*; Pth1r*^*f/+*^). Three-month-old male mice of the three genotypes were subjected to daily PTH1-34 injection for 28 d. Although either *Dmp1-Cre; Kindlin-2*^*f//+*^ or *Dmp1-Cre; Pth1r*^*f//+*^ mice did not display marked osteopenia compared to their Cre-negative controls (data not shown), interestingly, we found that the double heterozygous mice (*Dmp1-Cre; Kindlin-2*^*f/+*^*; Pth1r*^*f/+*^) displayed marked osteopenia with significant reductions in BMD, BV/TV, Tb.N, and Tb.Th and increase in Tb.Sp, when compared to those in either singly heterozygous mice (Fig. 3a–d and supplementary Fig. 3). PTH similarly increased BMD, BV/TV, Tb.N, Tb.Th, and Cort.Th and decreased Tb.Sp in both singly heterozygous mice (Fig. 3a–d and supplementary Fig. 3). More importantly, the PTH-induced increases in BMD, BV/TV, Tb.N, Tb.Th, and Cort.Th and decrease in Tb.Sp in both singly heterozygous mice were drastically reduced (BMD and BV/TV) or completely lost (Tb.N, Tb.Th, Cort.Th, and Tb.Sp) in double heterozygous mice (Fig. 3a–d and supplementary Fig. 3). Similar results were obtained in singly or double heterozygous female mice (supplementary Fig. 4). PTH treatment did not markedly impact animal body weight among these groups (supplementary Fig. 5).

Double heterozygous mice displayed impaired osteoblast function, as demonstrated by reduced MAR and BFR compared to those in singly heterozygous mice (Fig. 3e–g). Furthermore, PTH-stimulated increases in MAR and BFR in both singly heterozygous mice were dramatically reduced (for BFR) or completely lost (for MAR) in double heterozygous mice (Fig. 3e–g). Double heterozygous mice displayed higher basal osteoclast formation than either singly heterozygous mice (Fig. 3h–j). PTH increased osteoclast formation in both singly heterozygous bones, but not in double heterozygous mice (Fig. 3h–j). IF staining of osterix revealed a number of osteoblasts, which were dramatically increased by PTH, in bones of both singly heterozygous mice (Fig. 1k). Apparently, the numbers of osteoblasts were dramatically decreased in bones of doubly heterozygous mice, more importantly, which were not further increased by PTH (Fig. 1k).

Collectively, results from these experiments provide first evidence that Kindlin-2 and PTH1R function in the same genetic pathway in control of basal and PTH-stimulated osteoblast formation and bone mass.

### Kindlin-2 regulates endogenous PTH signaling in osteoblastic cells in vitro and in bone

**Fig. 4:**
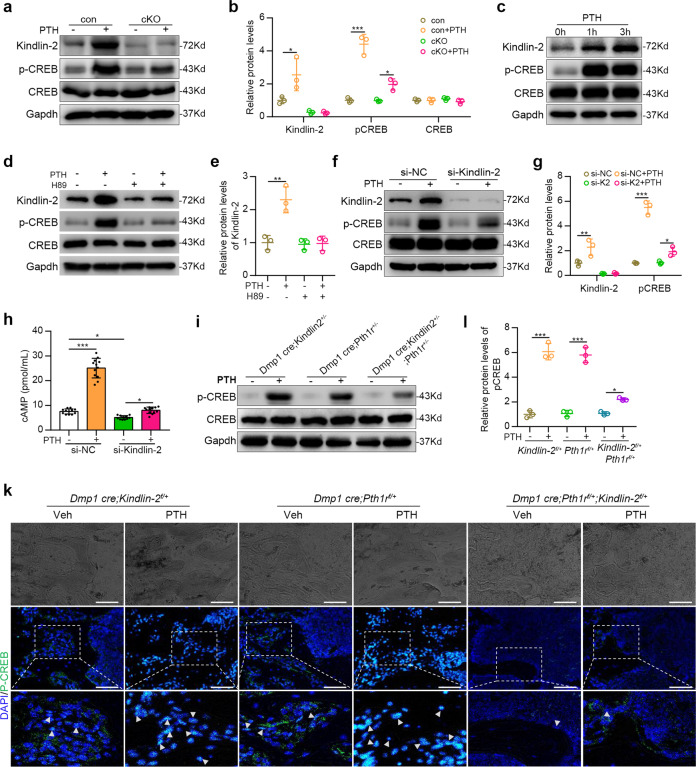
Kindlin-2 regulates PTH/PTH1R signaling in vitro and in bone. **a** Western blotting. Freshly prepared osteocyte-enriched mid diaphyseal femoral shafts (with their bone marrow flushed) of 3-month-old control (con) and cKO female mice were cut into tiny pieces and cultured in 10% FBS/α-MEM medium and treated with or without PTH 1-34 (10^−7^ M) for 3 h, followed by western blotting. **b** Quantitative analysis of protein expression of **a**. All experiments (**a**–**j**) were repeated three times independently. Results are expressed as mean ± standard deviation (s.d.). *P < 0.05, **P < 0.01, ***P < 0.001. **c** Western blot analysis. MC-4 cells were treated with PTH 1-34 (10^−7^ M) for the indicated times. **d** PKA inhibition. MC-4 cells were treated with and without PTH 1-34 (10^−7^ M) in the presence and absence of 10 μM H89 for 3 h. **e** Quantitative analysis of prot**e**in expression of **d**. **f** siRNA knockdown. MC-4 cells were transfected with control siRNA (si-NC) or si-Kindlin-2 (si-K2) and then treated with PTH 1-34 (10^−7^ M) for 3 h. **g** Quantitative analysis of protein expression of **f**. **h** siRNA knockdown. MC-4 cells were transfected with control siRNA (si-NC) or si-Kindlin-2 (si-K2) and then treated with PTH 1-34 (10^−7^ M) PTH for 3 h. cAMP concentration was measured using an ELISA kit. **i** Western blotting. Freshly prepared osteocyte-enriched mid diaphyseal femoral shafts (with their bone marrow flushed) of 3-month-old singly heterozygous and double heterozygous male mice were cultured in 10% FBS/α-MEM medium and treated with or without PTH 1-34 (10^−7^ M) for 3 h in vitro. **j** Quantitative data of **i**. **k** Immunofluorescence (IF) staining. Sections of tibial sections were subjected to IF staining with p-CREB antibody. Scale bars: 50 μm. Arrowheads indicate p-CREB (+) cells

Based on above in vivo results, we wondered whether Kindlin-2 directly regulates the PTH/PTH1R signaling in osteoblastic cells and in bone. We prepared fresh mid diaphyseal femoral shafts (with their bone marrow flushed) from 3-month-old control and dKO mice. These osteocyte-enriched shafts were cut into tiny pieces, cultured in 10% FBS/α-MEM media and treated with and without PTH1-34 (10^−7^ M) for 3 h, followed by preparation of protein extracts and western blotting for expression of Kindlin-2, phosphorylated CREB (p-CREB) and total CREB proteins. Using this ex vivo bone tissue culture model system, we were able to show that acute PTH treatment rapidly and strongly induced the phosphorylation of CREB without affecting its total protein level in control bone (Fig. 4a, b). The PTH-stimulated CREB phosphorylation was dramatically reduced in cKO bone (Fig. 4a, b). Interestingly, PTH increased the protein level of Kindlin-2 in bone (Fig. 4a, b). Likewise, PTH induced CREB phosphorylation in the murine MC-4 osteoblastic cells (Fig. 4c), which was decreased by PKA inhibition (Fig. 4d, e) or siRNA knockdown of Kindlin-2 (Fig. 4f, g). PTH-stimulated cAMP production was abolished by Kindlin-2 knockdown in MC-cells (Fig. 4h). Similar to results from bone, PTH also increased expression of Kindlin-2 in MC-4 cells. Finally, PTH induced CREB phosphorylation in singly heterozygous bones, which was significantly decreased in double heterozygous bones (Fig. 4i, j). IF staining of bone sections revealed some p-CREB (+) cells, as expected, which were stimulated by PTH, on bone surfaces (osteoblast) and in the bone matrix (osteocytes) in both *Dmp1-Cre; Kindlin-2*^*f//+*^ and, to a larger extent, *Dmp1-Cre; Pth1r*^*f//+*^ bones. Few p-CREB (+) cell were observed in bones of cKO mice with and without PTH treatment (Fig. 4k).

### Kindlin-2 interacts with the C-terminal cytoplasmic domain of PTH1R and the stimulatory subunit of G protein, Gsα, in osteoblastic cells or in COS-7 cells overexpressing both factors

**Fig. 5:**
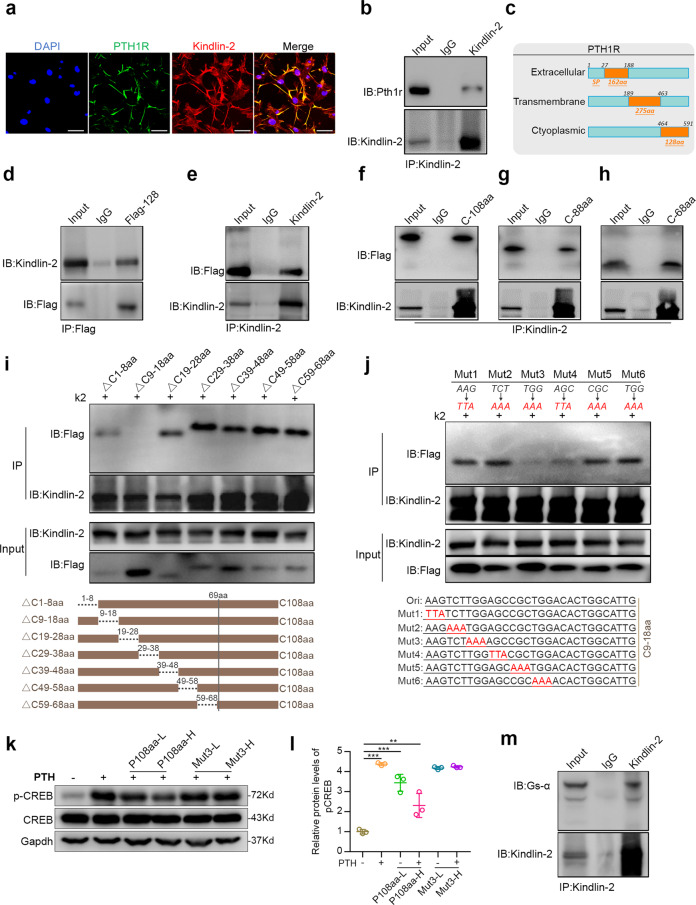
Kindlin-2 interacts with the C-terminal intracellular domain of PTH1R at aa 474–475 and Gsα. **a** Immunofluorescence (IF). Colocalization of Kindlin-2 and PTH1R in MLO-Y4 osteocyte-like cells. Scale bars: 20 μm. **b** Co-immunoprecipitation (co-IP) assay. Protein extracts from MC-4 cells were incubated with Kindlin-2 antibody or IgG, and the immunocomplexes were separated by SDS-PAGE, followed by western blotting with antibodies against PTH1R and Kindlin-2. **c** A diagram of the mouse PTH1R protein structure topology showing a signal peptide (SP, aa 1–27), extracellular domain (aa 28–188), transmembrane domain (aa 189–463), and cytoplasmic domain (aa 464–591). **d**, **e** co-IP assay. COS-7 cells were co-transfected with expression plasmids expressing Flag-PTH1R (128 aa) and full-length Kindlin-2. Protein extracts were incubated with either Flag antibody (**d**) or Kindlin-2 antibody (**e**). **f**–**h** co-IP assay. Truncated expression plasmids expressing PTH1R C-terminal cytoplasmic 108, 88, or 68 aa regions and full-length Kinldin-2 expression plasmid were co-transfected into COS-7 cells. Protein extracts were incubated with Kindlin-2 antibody, followed by western blotting using Flag and Kindlin-2 antibodies. **i** co-IP assay. Using the 108 aa plasmid as parent plasmid to generate seven internal deletion plasmids with 10 aa deleted in each. Different internal deletion plasmids and full-length Kindlin-2 plasmid were co-transfected into COS-7 cells. Protein extracts were incubated with Kindlin-2 antibody, followed by western blotting using Flag and Kindlin-2 antibodies. **j** co-IP assay. Using the 108 aa plasmid as parent plasmid, six plasmids with point mutations within the C9–18 aa region were generated and co-transfected with full-length Kindlin-2 expression plasmid in COS-7 cells. Proteins extracts were incubated with Kindlin-2 antibody, followed by western blotting using Flag and Kindlin-2 antibodies. **k** Western blotting. MC-4 cells were transfected with low (800 ng, L) and high dose (1600 ng, H) of wild-type PTH1R108 aa (P108 aa) or mutant PTH1R108 aa (Mut3) plasmids and then treated with PTH 1-34 (10^−7^ M) for 3 h. Whole-cell lysates were collected for western blotting using the indicated antibodies. **l** Quantitative analysis of protein expression of **k** from three independent experiments. Results are expressed as mean ± standard deviation (s.d.). **P < 0.01, ***P < 0.001. **m** co-IP assay. Protein extracts from MC-4 cells were incubated with Kindlin-2 antibody or IgG, and the immunocomplexes were separated by SDS-PAGE, followed by western blotting with Gsα or Kindlin-2 antibody

To explore mechanisms through which Kindlin-2 mediates the PTH actions, we next determined whether Kindlin-2 interacts with PTH1R. As an initial step, we performed immunofluorescence (IF) staining in the MLO-Y4 osteocyte-like cells. The results showed that PTH1R was localized at the plasma membrane, while Kindlin-2 was widely distributed all over the whole cell. A strong colocalization of both factors was observed on the cell membrane (Fig. 5a). We next performed co-immunoprecipitation (co-IP) assays using whole-cell extracts isolated from the murine MC-4 osteoblastic cells, which express high levels of both factors (Fig. 5b) and found that PTH1R protein was present in the Kindlin-2 immunoprecipitates (Fig. 5b), thus demonstrating an interaction of the endogenous Kindlin-2 and PTH1R proteins.

As a seven transmembrane protein, PTH1R possesses a relatively large N-terminal extracellular domain (aa 27–187), three extracellular and three cytoplasmic loops and a 128 aa C-terminal cytoplasmic region (aa 464–591). Because Kindlin-2 is a cytoplasmic protein, we wondered whether Kindlin-2 can interact with the C-terminal cytoplastic domain of PTH1R (Fig. 5c). Kindlin-2 and Flag-tagged cytoplasmic domain of PTH1R were co-expressed in COS-7 cells. Whole-cell extracts were prepared for co-IP assays. The results revealed that Kindlin-2 was present in the Flag immunoprecipitates (Fig. 5d) and, vice versa, Flag-tagged 128-aa C-terminal cytoplasmic domain protein was detected in the Kindlin-2 immunoprecipitates (Fig. 5e).

We next performed several sets of experiments to identify potential binding sites within PTH1R molecule responsible for Kindlin-2 interaction. We generated several plasmids expressing the aa 1–108, aa 1–88, and aa 1–68 regions of the cytoplasmic domain and co-transfected them with Kindlin-2 expression plasmid in COS-7 cells, followed by co-IP assays. The results showed that deletion from aa 128 to aa 69 did not markedly affect Kindlin-2 interaction (Fig. 5f–h), suggesting that the Kindlin-2-interacting domain is located in the aa 1–68 region of the cytoplasmic domain. To further define a smaller region necessary for Kindlin-2 interaction, we next used the PTH1R aa 1–108 plasmid as parent plasmid and generated 7 internal deletion plasmids; each contained 8 or 10 aa residues as indicated and, thus, fully covered the whole aa 1–68 region. We then co-transfected these internal deletions plasmids with Kindlin-2 expression plasmid in COS-7 cells, followed by co-IP assays. Results showed that deleting the aa 9–18 region completely abolished the Kindlin-2 interaction (Fig. 5i).

To further identify aa residue(s) responsible for Kindlin-2 interaction, we used the PTH1R aa 1–108 plasmid as a parent plasmid and generated a series of expression plasmids with the indicated point mutations (Fig. 5j). The results from co-IP experiments using these constructs revealed that Kindlin-2 interaction was essentially abolished by mutation #3 (Mut3), in which TGG (Trp474) was changed to AAA (Lys474) (Fig. 5j). Mutation #4 (Mut4), which converted AGC (Ser475) to TTA (Leu475), also markedly reduced Kindlin-2 interaction (Fig. 5j). Thus, residues 474–475 of PTH1R are critical for Kindlin-2 interaction.

We next determined the effects of the overexpression of a cytoplasmic region of PTH1R on endogenous PTH1R signaling in MC-4 cells. In this experiment, cells were transfected with plasmids expressing wild-type (WT) or mutant (Mut3) PTH1R aa 1–108 region and then treated with and without PTH1-34 (10^−7^ M) for 3 h. Results from western blotting showed that overexpression of the WT PTH1R aa 1–108 region dose-dependently reduced the PTH-stimulated CREB phosphorylation (Fig. 5k, l). In contrast, overexpression of the Kindlin-2-interacting-deficient mutant (Mut3) PTH1R aa 1–108 region failed to reduce the PTH-induced CREB phosphorylation in MC-4 cells (Fig. 5k, l).

We next determined whether Kindlin-2 interacts with Gsα, a major downstream effector of PTH1R. Protein extracts from MC-4 cells were incubated with Kindlin-2 antibody or IgG, and the immunocomplexes were separated by SDS-PAGE, followed by western blotting with Gsα or Kindlin-2 antibody. The result showed that both Gsα or Kindlin-2 proteins were present in the Kindlin-2 immunoprecipitates (Fig. 5m).

### Estrogen deficiency dramatically reduces Kindlin-2 expression in osteocytes and Kindlin-2 loss completely abolishes intermittent PTH-stimulated bone volume and BMD in ovariectomized mice

**Fig. 6:**
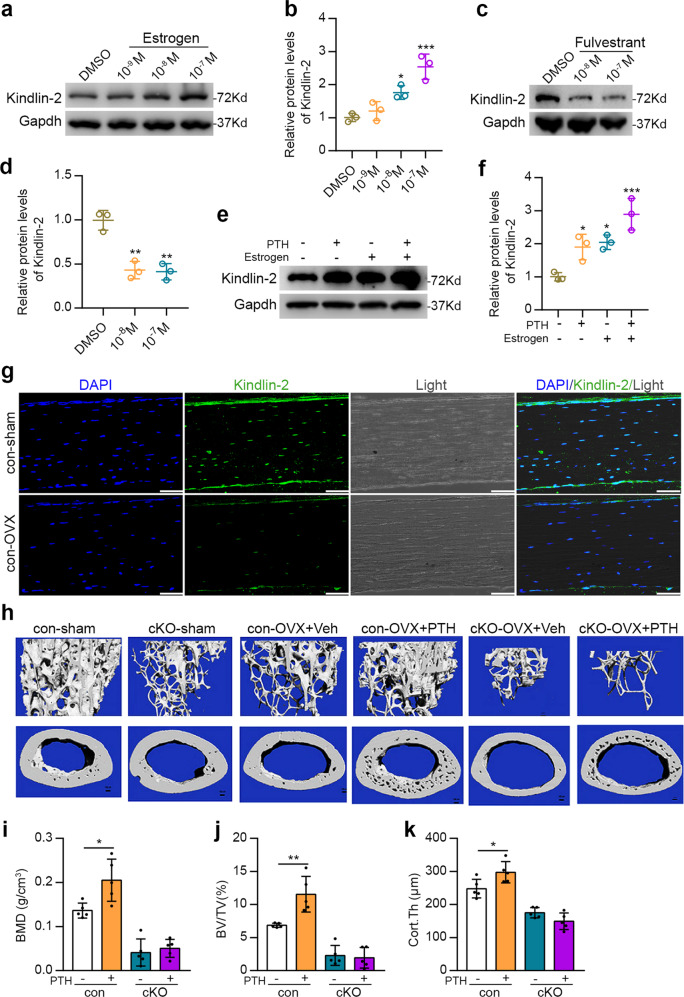
Estrogen deficiency decreases Kindlin-2 expression in osteocytes and Kindlin-2 loss abolishes intermittent PTH-stimulated bone mass in OVX mice. **a**–**f** Western blot analysis. MLO-Y4 cells were treated with increasing concentration of estrogen (**a**), fulvestrant (**c**), or PTH and/or estrogen (**e**) for 3 h. Gapdh was used as a loading control. Quantitative data from three independent experiments (**b**, **d**, **f**). Results are expressed as mean ± standard deviation (s.d.). *P < 0.05, **P < 0.01, ***P < 0.001, versus veh. **g** Immunofluorescence staining of tibial sections of control-sham and control-OVX mice with an anti-Kindlin-2 antibody, Scale bars: 50 μm. **h** Representative micro-CT (μCT) images of distal femurs. Four-month-old control and cKO mice were subjected to sham and OVX operation. Two months later, mice were administrated with PTH (100 μg/kg) for 28 d. **i**–**k** Quantitative analyses of the bone volume/tissue volume (BV/TV), bone mineral density (BMD), and cortical thickness (Cort.Th) of distal femurs from control and cKO mice. *N* = 5 mice per group. Results are expressed as mean ± standard deviation (s.d.). *P < 0.05, **P < 0.01, versus veh

In an effort to identify factors that regulate expression of Kindlin-2 in osteoblastic cells, we found that estrogen significantly increased the level of Kindlin-2 protein in MC-4 cells in a dose-dependent manner (Fig. 6a, b). In contrast, fulvestrant, an estrogen receptor antagonist, dramatically decreased Kindlin-2 expression in MC-4 cells (Fig. 6c, d). We did not observe a synergistic effect of PTH and estrogen on stimulation of expression of Kindlin-2 in MC-4 cells (Fig. 6e, f). We next determined whether estrogen deficiency reduces Kindlin-2 expression in bone and the effects of intermittent PTH on bone by using the ovariectomized (OVX) mouse model. To this end, 4-month-old control and cKO female mice were subjected to ovariectomy or sham surgery as we previously described.^34^ Two months after surgery when the bone mass was reduced, mice were subjected to daily PTH 1-34 injection (100 μg/kg body weight) for 28 d. Consistent with results from in vitro experiments, estrogen deficiency induced by OVX drastically decreased the expression of Kindlin-2 in osteocytes embedded in the bone matrix (Fig. 6g). Strikingly, Kindlin-2 deletion completely abolished the PTH-stimulated increases in BV/TV, BMD, and Cort.Th in OVX mice (Fig. 6h–k).

## Discussion

In this study, we demonstrate that Kindlin-2 regulates PTH1R in osteoblastic cells to control skeletal mass accrual and homeostasis and promote the anabolic effects of intermittent PTH on bones by modulating osteoblast and osteoclast formation. Thus, we define a previously unknown function of Kindlin-2 and a distinct regulatory mechanism of the PTH/PTH1R signaling in bone. Our findings may provide a therapeutic strategy for metabolic bone diseases, such as osteoporosis.

This is the first demonstration that Kindlin-2, a key focal adhesion protein, through its expression in osteoblastic cells, regulates the PTH anabolic activity in bone. Thus, deleting Kindlin-2 expression in these cells dramatically decreases the intermittent PTH-stimulated increases in bone volume and BMD in both male and female mice. Our results suggest that Kindlin-2 exerts such a function by modulating PTH1R that is mainly expressed in cells of the osteoblastic lineage. This notion is strongly supported by the following lines of evidence from the present study and from literature: (1) It is known that PTH functions primarily through PTH1R; (2) deleting one allele of *Kindlin-2* and *Pth1r* genes, but not one allele of ether gene, severely impairs the PTH anabolic activity in bone in both male and female mice, demonstrating that both factors function in the same genetic pathway in bone; (3) Kindlin-2 loss decreases the PTH-stimulated CREB phosphorylation in bone, a major downstream event of PTH/PTH1R signaling; (4) siRNA knockdown of Kindlin-2 expression reduces PTH-stimulated CREB phosphorylation and cAMP production in osteoblastic cells; (5) Kindlin-2 interacts with the C-terminal cytoplasmic domain of PTH1R in MC-4 cells and in COS-7 cells expressing both factors; and (6) Overexpression of a WT, but not a Kindlin-2-interacting-deficient (Mut3), cytoplasmic region of PTH1R inhibits endogenous PTH/PTH1R signaling in MC-4 cells. In addition, Kindlin-2 also interacts with Gsα, the stimulatory subunit of G protein. Because Gsα directly binds to PTH1R and transduces PTH/PTHrP signaling, which further activates protein kinase A and other signaling pathways, it is possible that Kindlin-2 stabilizes the PTH1R-Gsα complex by interacting with both factors and thereby promotes PTH/PTHrP signaling in osteoblastic cells. Notably, Fulzele et al. recently reported that deleting Gsα expression utilizing the same *Dmp1-Cre* used in the present study led to osteopenia due to sclerostin-induced suppression of osteoblast activity.^35^

It is important to note that the *Kindlin-2* and *Pth1r* double heterozygous mice (*Dmp1-Cre; Kindlin-2*^*f/+*^*; Pth1r*^*f/+*^) also display significant reductions in basal bone volume and BMD, when compared to singly heterozygous mice (*Dmp1-Cre; Kindlin-2*^*f//+*^ or *Dmp1-Cre; Pth1r*^*f/+*^). This suggests that the interplay between Kindlin-2 and PTH1R plays an important role in control of skeletal homeostasis under normal physiological condition. Thus, our findings of the present study are of broad significance.

It is widely believed that cells of the osteoblastic lineage are the primary target of intermittent PTH. Intermittent PTH also stimulates osteoclast formation in bone. In fact, osteoclast formation is essential for the anabolic activity of PTH in bone.^13,36^ Interestingly, Kindlin-2 and PTH1R cooperatively regulate not only the osteoblast-mediated bone formation, but also the osteoclast formation in bone. While Kindlin-2 loss in osteoblastic cells increases the basal level osteoclast formation in bone, which is consistent with our previously published results,^30^ it abolished the intermittent PTH stimulation of osteoclast formation in bone, which can be partially explained by the fact that intermittent PTH induces expression of RANKL in control but not cKO bones.

We find that estrogen regulates Kindlin-2 expression in cultured osteoblastic cells, while systematic estrogen deficiency by OVX in mice drastically decreases the expression of Kindlin-2 protein in osteocytes embedded in the mineralized bone matrix. Unexpectedly, Kindlin-2 loss completely abolished the PTH anabolic activity in bone in OVX mice.

Findings of the present study raise a possibility that the focal adhesion activation by ECM signals favors skeletal development and homeostasis by facilitating PTH signaling through promotion of the Kindlin-2-PTH1R interaction. Future study will explore this interesting possibility.

**Fig. 7:**
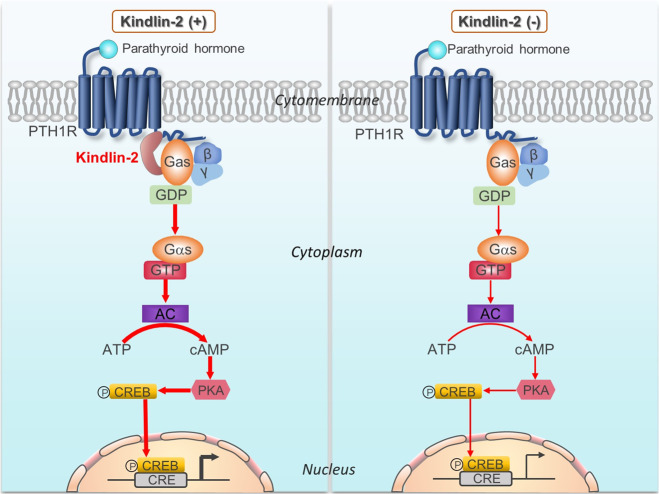
Working model

Based on findings from this and other studies, we propose that Kindlin-2 functions as an intrinsic component of the PTH/PTHrP/PTH1R signaling pathway in osteoblastic cells to regulate bone mass accrual and homeostasis (Fig. 7). Kindlin-2 interacts with the cytoplasmic domain of PTH1R via aa 474–475 and Gsα, the stimulatory subunit of G protein, thus facilitating PTH signaling and thereby osteoblast and osteoclast formation/function and bone homeostasis. In the meantime, PTH increases expression of Kindlin-2, thus creating a positive feedback regulatory loop (not shown). In the absence of Kindlin-2, PTH signaling in osteoblastic cells is weakened and fails to increase bone mass. Furthermore, estrogen upregulates expression of Kindlin-2 in osteoblastic cells (not shown), which may contribute to the PTH anabolic activity in bone.

Results from the present study highlight a requirement to investigate whether loss of Kindlin-2 in osteoblastic cells plays a role in the pathogenesis of metabolic bone diseases, such as aging- and menopause-associated osteoporosis.

## Materials and methods

### Animal studies

The generation of the *Kindlin-2*^*fl/fl*^ mice was recently described.^24^ *Dmp1-Cre* transgenic mice, in which a fragment of the mouse dentin matrix protein 1 (*Dmp1*) gene promoter drives Cre recombinase expression in osteocytes, were described.^37^ The generation of the *Dmp1-Cre; Kindlin-2*^*f/f*^ mice was previously described.^30^ *Pth1r*^*f/f*^ mice were previously described,^5^ generously provided by Dr. Meiqing Wang (the Fourth Military Medical University, Xi’an, China)^38^ and bred to *Dmp1-Cre; Kindlin-2*^*f/f*^ mice to generate *Dmp1-Cre; Kindlin-2*^*f/+*^, *Dmp1-Cre; Pth1r*^*f/+*^, *Dmp1-Cre; Kindlin-2*^*f/+*^*; Pth1r*^*f//+*^ mice and other genotypes for this study. Mice were subjected to daily subcutaneous administration of PTH 1-34 (Bachem) (100 μg/kg body weight) for 28 d. For acute effect of PTH on protein expression in bone, cortical bone shafts were cut into tiny pieces and treated with PTH 1-34 (10^−7^M) for 3 h, followed by western blotting using the indicated antibodies. Mice were housed 4–6 mice/cage at 20–24 °C and exposed to a 12-h/12-h light-dark period.

### Micro-computerized tomography (μCT) analysis

Fixed non-demineralized femurs were used for micro-computerized tomography (μCT) analysis in the Department of Biology of Southern University of Science and Technology using a Bruker μCT (SkyScan 1172 Micro-CT, Bruker MicroCT, Kontich, Belgium) following the standards of techniques and terminology recommended by the American Society for Bone and Mineral Research (ASBMR).^39^ Obtained slices were reconstructed using NRecon software (NRecon), CTAn was used to analyze the parameters of trabecular and cortical bone. Three-dimensional model was constructed by software (CTvox). Contours were defined and drawn close to the cortical bone. VOIs of trabecular bone selected for analysis was a 1.5-mm length of the metaphyseal secondary spongiosa, originating 0.5 mm below the epiphyseal growth plate and extending caudally. A 1.0-mm section was used to obtain midfemoral cortical bone thickness. The analysis of the specimens involved the following bone measurements: the bone volume/tissue volume fraction (BV/TV), trabecular number (Tb.N), trabecular separation (Tb.Sp), trabecular thickness (Tb.Th), cortical thickness (Cort.Th), and bone mineral density (BMD).

### Calcein double labeling, mineral apposition rate (MAR), mineralizing surface per bone surface (MS/BS), and bone formation rate (BFR)

Mice were injected i.p. with calcein (20 mg/kg) at 6 and 2 days before sacrifice. Non-demineralized tibiae were embedded using an Osteo-Bed Bone Embedding kit (EM0200; MilliporeSigma) and sectioned at 5 μm. Images were captured using a fluorescence microscope (Olympus-BX53). MAR, MS/BS, and BFR were measured as previously described.^40^

### Osteoclast formation in bone

The TRAP staining of bone sections was performed as we previously described.^41^ The osteoclast surface and osteoclast number were determined as we previously described.^42^

### ELISA assays

ELISA assay was performed as previously described.^26^ MC-4 cells transfected with si-NC or si-Kinldin-2 for 36 h were treated with PTH for 3 h. Whole-cell lysates were prepared in RIPA lysis buffer (150 mM NaCl, 1% NP-40, 50 mM Tris, 5 mM NaF, and 0.1% SDS). Quantitative determination of cyclic AMP (cAMP) in cell lysates was performed using the cAMP Assay Kit (R&D Systems) according to the manufacturer’s instruction.

### Western blot analysis

Western blot analysis was performed as previously described.^43^ Briefly, whole-cell lysates were prepared in RIPA lysis buffer (150 mM NaCl, 1% NP-40, 50 mM Tris, 5 mM NaF, and 0.1% SDS) and aliquots of 30 μg protein were separated by SDS-PAGE and blotted onto a polyvinylidene fluoride (PVDF) membrane (Millipore, MA, USA). Membranes were blocked at room temperature For 1 h in 5% non-fat powdered milk in Tris-buffered saline, followed by an overnight incubation at 4 °C with specific antibodies. After incubation with appropriate HRP-conjugated secondary antibodies (Santa Cruz), blots were developed using an enhanced chemiluminescence (ECL Kit, Millipore) and exposed in ChemiDoc XRS chemiluminescence imaging system (Bio-Rad). Antibodies used in this study are listed in supplementary Table 1.

### Quantitative real-time RT-PCR and western blot analyses

RNA isolation and quantitative real-time RT-PCR analysis were performed as previously described.^44^ The specific primers for gene expression analysis were listed in supplementary Table 2.

### Co-immunoprecipitation assay

Co-immunoprecipitation assay was performed as previously described.^31^ Briefly, Cells were transfected with corresponding expression plasmids. After 24 h, cells were incubated for 10 min at 4 °C in IP lysis buffer (pH 7.4, 0.025 M Tris, 0.15 M NaCl, 0.001 M EDTA, 1% NP-40, and 5% Glycerol) (Thermo Fisher) containing proteinase inhibitor cocktail. After a centrifugation at 13,000 × *g* for 10 min at 4 °C, the supernatant was first incubated with corresponding primary antibody overnight and then with Protein A/G Magnetic Beads at room temperature for 1 h. DynaMag™-2 Magnet (Thermo Fisher) was used to collect dynabeads-antigen-antibody complex. The complex was washed with IP buffer three times and resuspended with 60 μl 1× loading buffer and cooked at 95 °C for 5 min, followed by SDS-PAGE and western blotting.

### Immunofluorescence staining

Immunofluorescence staining was performed as previously described.^30^ Briefly, cells grown on confocal dish (SPL life science) were washed in 1× PBS three times and then fixed in 4% PFA for 10 min. After permeabilization with 1% Triton X-100 for 15 min and incubation in the blocking solution (1% BSA) for 1 h at room temperature, cells were incubated with the corresponding primary antibodies overnight at 4 °C, followed by incubation with secondary antibodies conjugated with Alexa 488, 568 for 1 h at room temperature. The cells were washed and then counterstained with 4’,6-diamidino-2-phelylindole (DAPI). Cells were visualized at 40x under SP8 lighting confocal microscopy (Leica) and images collecting software was LAS (Leica).

### Flow cytometry and single-cell RNA-sequencing library construction and scRNA-seq data analysis

Depletion of the hematopoietic cells from pooled whole BM preparations (three mice per group) was performed using the Lineage Cell Depletion Kit (cat. no. 130-090-858; Miltenyi Biotec, Auburn, CA) according to manufacturer’s instructions. Cells (DAPI^-^; Calcein-AM^+^; CD45^−^; TER119^−^; CD71^−^ cells) sorted out by flow cytometry were then used for single-cell RNA sequencing based on 10X Genomics Chromium Single Cell 3’ protocol (v3 Chemistry). Quality control and quantification was performed using Agilent 4200 TapeStation System (Agilent Technologies, California) and QuantStudio 5 Real-Time PCR System (Thermo Fisher Scientific, Massachusetts). Sequencing (PE 150 bp) was completed by NovaSeq 6000 Sequencing System (Illumina, California). Raw sequencing data were processed with Cell Ranger (v3.0.1) to align reads and generate feature-barcode matrices. Reference genome data of mm10 (v3.0.0) was downloaded from 10X Genomics. Raw feature-barcode matrices in Cell Ranger output were used for Seurat (v3.1.0) analysis.^45^ Initially, cells with extremely low detected genes (<30) or genes detected in few cells (<5) were excluded while Seurat object was created. Then, 3000 variable features (genes) were used for principle component analysis (PCA). A shared nearest neighbor (SNN) was constructed based on the top 30 PCs, and clusters of cells were identified by this SNN modularity optimization-based clustering algorithm with resolution at 2. Quality control was firstly based on feature counts in each cluster, and clusters with relative low mean feature counts (about 200) were removed. The filtered scRNA-seq data were then used to identify cluster again with same parameters. Clusters with relative low mean feature counts (about 200) and expressing hematopoietic specific genes including Hba-a1 (red blood cell), Pf4 (platelet), Cd45 (immune cells) were excluded, remaining clusters were then processed again for clustering. For cells in each cluster, we removed top 5% and bottom 5% by feature counts, and top 5% by counts percentage of mitochondrial genes. Seurat objects of four samples were merged into one for combination analysis. We selected top 3000 variable features, and 50 PCs were computed, then top 30 of them were used for constructing SNN, clusters were identified with resolution at 1. Uniform Manifold Approximation and Projection (UMAP) was enrolled for data visualization.^46^ Marker genes of each cluster with average log fold change higher than 0.8 were used for plotting heatmap and calculate Pearson’s correlation between clusters. Cell number used for heatmap was no more than 50 for each cluster. Pearson’s correlation was based on average expression level in each cluster. Differential gene expression analysis was performed between samples for each cluster. Gene ontology enrichment analysis was performed using significantly differentially expressed genes with Metascape.^47^

### Statistical analyses

The sample size for each experiment was determined based on our previous experience in similar studies. Mice used in experiments of this study were randomly grouped. IF and histology were performed and analyzed in a double-blinded manner. Statistical analyses were completed using the Prism GraphPad. One-way ANOVA with Tukey’s multiple-comparisons test was used unless specifically indicated. *P* < 0.05 was considered statistically significant

### Study approval

All animal experiments were approved by the Institutional Animal Care and Use Committee (IACUC) of Southern University of Science and Technology. We affirm that we have all relevant ethical regulations for animal testing and research in this study.

## Supplementary information

Supplemental information

## Data Availability

All data generated for this study are available from the corresponding authors upon reasonable request.
