# Supplementary material for: Kindlin-2 regulates skeletal homeostasis by modulating PTH1R in mice
Source: Signal Transduct Target Ther. 2020 Dec 26;5:297. doi: 10.1038/s41392-020-00328-y (PMC7762753; doi:10.1038/s41392-020-00328-y)
Supplement: Supplementary file 1 — Supplemental information [file 41392_2020_328_MOESM1_ESM.docx]

Supplementary Materials for

Kindlin-2 regulates skeletal homeostasis by modulating PTH1R in mice

Xuekun Fu, Bo Zhou, Qinnan Yan, Chu Tao, Lei Qin, Xiaohao Wu, Sixiong Lin, Sheng Chen, Yumei Lai, Xuenong Zou, Zengwu Shao, Meiqing Wang, Di Chen, Wenfei Jin, Youqiang Song, Huiling Cao, Ge Zhang, Guozhi Xiao

Correspondence to: xiaogz@sustech.edu.cn

This PDF file includes:

Supplemental Figure 1

Supplemental Figure 2

Supplemental Figure 3

Supplemental Figure 4

Supplemental Figure 5

Supplemental Figure 6

Supplementary Table 1: Antibody information

Supplementary Table 2: Mouse qPCR primers

**Supplementary information**

| **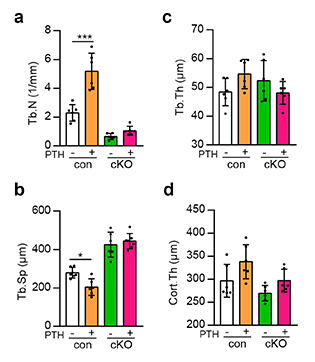** |
| --- |
| **Supplemental Figure 1. Kindlin-2 loss greatly impairs the anabolic effects of intermittent PTH on bone.** (**a**-**d**) Quantitative analyses of the trabecular number (Tb.N), trabecular separation (Tb.Sp), trabecular thickness (Tb.Sp) and cortical thickness (Cort.Th) of the distal femurs from 3-month-old control and cKO female mice. N = 6 per group. Results are expressed as mean ± standard deviation (s.d.). ***P* < 0.01, ****P* < 0.001, versus veh. |

| **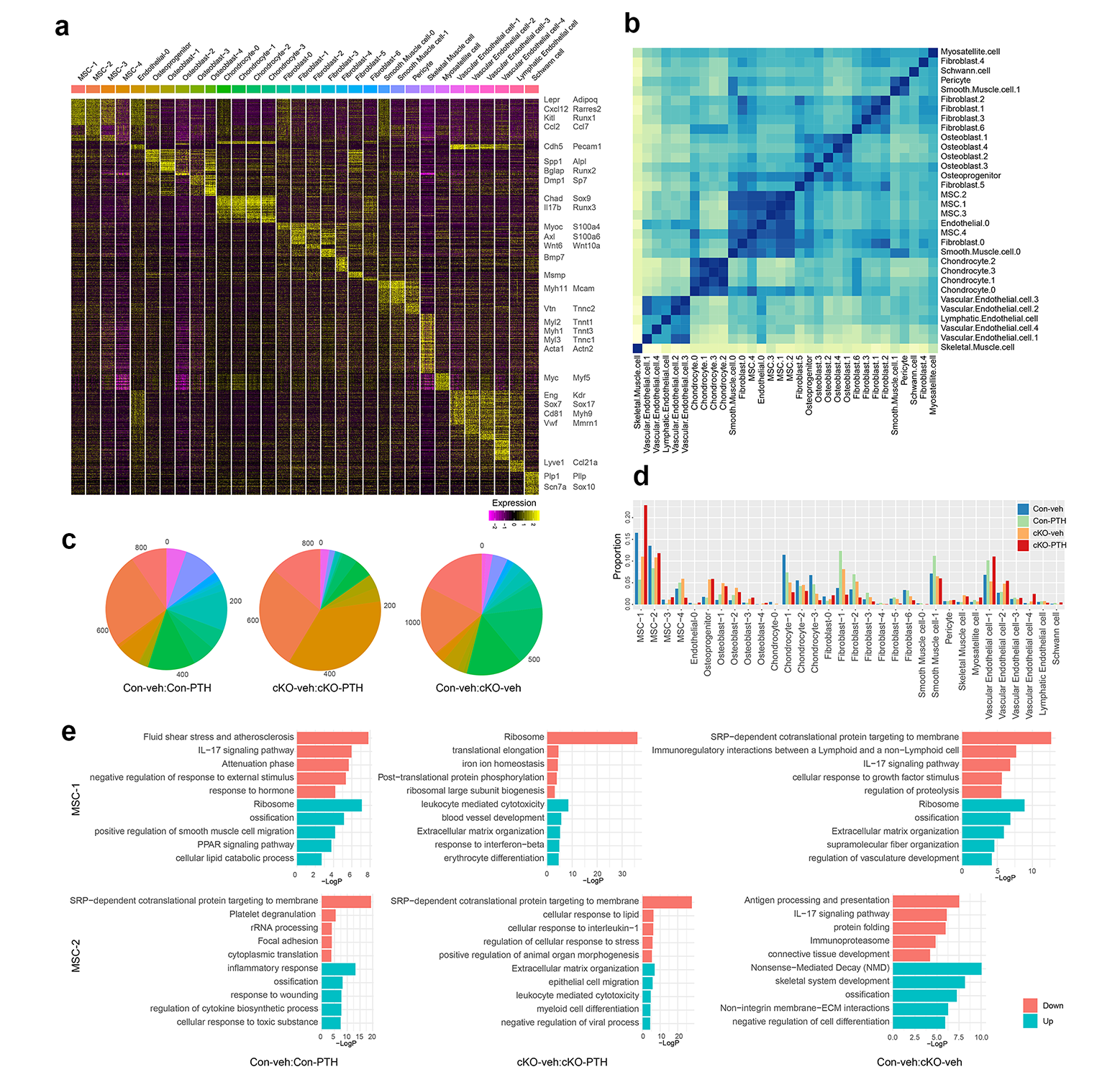** |
| --- |
| **Supplemental Figure 2. Single-cell RNA sequencing analysis.** (**a**) Heatmap of marker genes in each cluster. Cell number in each cluster was down sampled as 50, marker genes were selected as described in Methods. (**b**) Heatmap of Pearson’s correlation of each cluster. (**c**) Pie plot of significantly and differentially expressed genes in each cluster. Different clusters were colored as in (a). (**d**) Distribution of each cluster among samples. (**e**) Gene enrichment analysis of differentially expressed genes in MSC-1 and MSC-2, top 5 resulted for up-regulated genes and down-regulated genes were selected. |

| **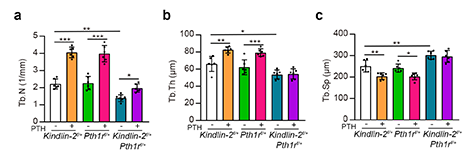** |
| --- |
| **Supplemental Figure 3. Double heterozygous male mice display striking osteopenia and impaired response to PTH.** (**a**-**c**) Quantitative analyses of the trabecular number (Tb.N), trabecular separation (Tb.Sp) and trabecular thickness (Tb.Th) of distal femurs from 3-month-old *Dmp1-Cre; Kindlin-2^f/+^*, *Dmp1-Cre; Pth1r^f/+^* and *Dmp1-Cre; Kindlin-2^f/+^; Pth1r^f/+^* male mice with and without PTH treatment for 28d at the age of 3 months. N = 6 mice per group. Results are expressed as mean ± standard deviation (s.d.). **P* < 0.05, ***P* < 0.01, ****P* < 0.001. |

| **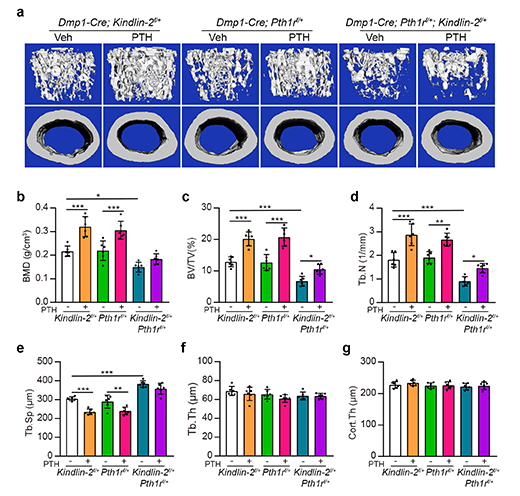** |
| --- |
| **Supplemental Figure 4. Double heterozygous female mice exhibit reduced bone mass and defective PTH response**. (**a**) Three-dimensional (3D) images of micro-computerized tomography (μCT) of distal femurs from *Dmp1-Cre; Kindlin-2^f/+^*, *Dmp1-Cre; Pth1r^f/+^* and *Dmp1-Cre; Kindlin-2^f/+^; Pth1r^f/+^* female mice with and without PTH treatment for 28d at the age of 3 months. (**b**-**g**) Quantitative analyses of the bone mineral density (BMD), bone volume/tissue volume (BV/TV), trabecular number (Tb.N), trabecular separation (Tb.Sp), trabecular thickness (Tb.Th), and cortical thickness (Cort.Th) of distal femur. N = 6 mice per group. Results are expressed as mean ± standard deviation (s.d.). **P* < 0.05, ***P* < 0.01, ****P* < 0.001. |

| **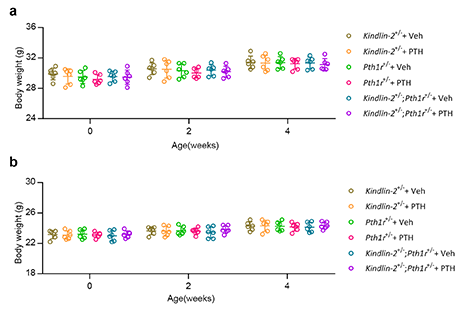** |
| --- |
| **Supplemental Figure 5. Growth curve.** Mouse growth curve for male (**a**) and female (**b**) mice during PTH injection. N = 6 per group. |

| **Supplementary Table 1: Antibody information** | | | |
| --- | --- | --- | --- |
| **Antibody** | **Company** | **Catalog#** | **Dilution** |
| Gapdh | Beyotime | AF0006 | WB (1:2000) |
| Kindlin-1 | Sigma | SAB4200465 | WB (1:1000) |
| Kindlin-2 | Proteintech | 11453-1-AP | WB (1:1000), IP (3 μg) |
| Kindlin-2 | Millipore | MAB2617 | WB (1:1000), IF (1:400) |
| Kindlin-3 | CST | 13843 | WB (1:1000) |
| CREB | CST | 9197S | WB (1:1000) |
| p-CREB | CST | 9198S | WB (1:500) |
| Pth1r | Sigma | SAB4502493 | IF (1:200) |
| Flag | Sigma | F1804 | WB (1:3000), IP (3 μg) |
| Osterix | Abcam | ab22552 | IF (1:200) |
| Gsα | Sigma | MABN543 | WB (1:1000) |

| **Supplementary Table 2: Mouse qPCR primers** | | |
| --- | --- | --- |
| **Name** | **5' primer** | **3' primer** |
| Actin | GGCTGTATTCCCCTCCATCG | CCAGTTGGTAACAATGCCATGT |
| Gapdh | AGGTCGGTGTGAACGGATTTG | TGTAGACCATGTAGTTGAGGTCA |
| Rankl | CAGCATCGCTCTGTTCCTGTA | CTGCGTTTTCATGGAGTCTCA |
| Opg | GGCTGAGTGTTTTGGTGGACAG | GCTGGAAGGTTTGCTCTTGTGA |
